# The role of affective temperaments in predicting symptom severity in bipolar disorder

**DOI:** 10.1192/j.eurpsy.2021.242

**Published:** 2021-08-13

**Authors:** M. Raia, C. Ciampi, F. Zinno, V. Caivano, E. Barone, G. Sampogna, V. Del Vecchio, V. Giallonardo, L. Steardo, M. Luciano, A. Fiorillo

**Affiliations:** 1 Department Of Psychiatry, University of Campania “Luigi Vanvitelli”, Naples, Italy; 2 Health Sciences, University Magna Graecia, Catanzaro, Italy

**Keywords:** bipolar disorder, temperament, symptom, Screening

## Abstract

**Introduction:**

Bipolar disorder (BD) is one of the most burdensome psychiatric illnesses, being associated with a negative long-term outcome and high suicide rate. Although affective temperaments are considered possible mediators of outcome, their role on the course and outcome of BD remains poorly studied.

**Objectives:**

The aims of the present study are to describe the clinical characteristics of patients with BD more frequently associated with the different affective temperaments and to verify which affective temperaments are associated with a more severe clinical picture in a sample of patients with BD.

**Methods:**

All patients with BD referring to the outpatient units of two Italian university sites have been recruited. Patients’ psychiatric symptoms, affective temperaments, and quality of life were investigated through validated assessment instruments.

**Results:**

199 patients were recruited. 54.8% of patients had a diagnosis of bipolar I disorder. 56.8% of the sample reported at least one episode of aggressive behaviours and 30.2% of suicidal attempt. Predominant cyclothymic and irritable temperaments predicted more frequent relapses, a poorer quality of life (p<;0.05), more aggressive behaviours and suicide attempts (p<;0.01). The predominant hyperthymic disposition was a protective factor for several outcome measures, including relapses and suicidality (p<;0.01), and was correlated with a less severity of psychiatric symptoms and later age at onset (p<;0.05).

**Conclusions:**

Early identification of affective temperaments in BD patients can help clinicians to identify those who could show a worse prognosis. A screening of affective temperaments can be useful to develop early targeted integrated pharmacological and psychosocial interventions.

**Disclosure:**

No significant relationships.

